# Supplementary material for: Genome-Wide Analysis of Terpene Synthase Genes in Crocus sativus Reveals Their Regulatory Roles in Terpenoid Biosynthesis and Abiotic Stress Tolerance
Source: Int J Mol Sci. 2025 Sep 30;26(19):9548. doi: 10.3390/ijms26199548 (PMC12524938; doi:10.3390/ijms26199548)
Supplement: Supplementary file 1 [file ijms-26-09548-s001.zip › Figure S1.docx]

**Supplementary Figure S1.** In Silico PCR Analysis for *CsTPS1*. (**A**) Virtual gel electrophoresis showing amplicon bands for Primer Pair 7 on a 2% agarose gel with a 100 bp ladder. (**B**) In silico cloning of *CsTPS1* into pET-28a (+), showing insertion/deletion sites for 6xHis-tag and stop codon. (**C**) uMelt melting curve for Primer Pair 7, confirming a single peak.


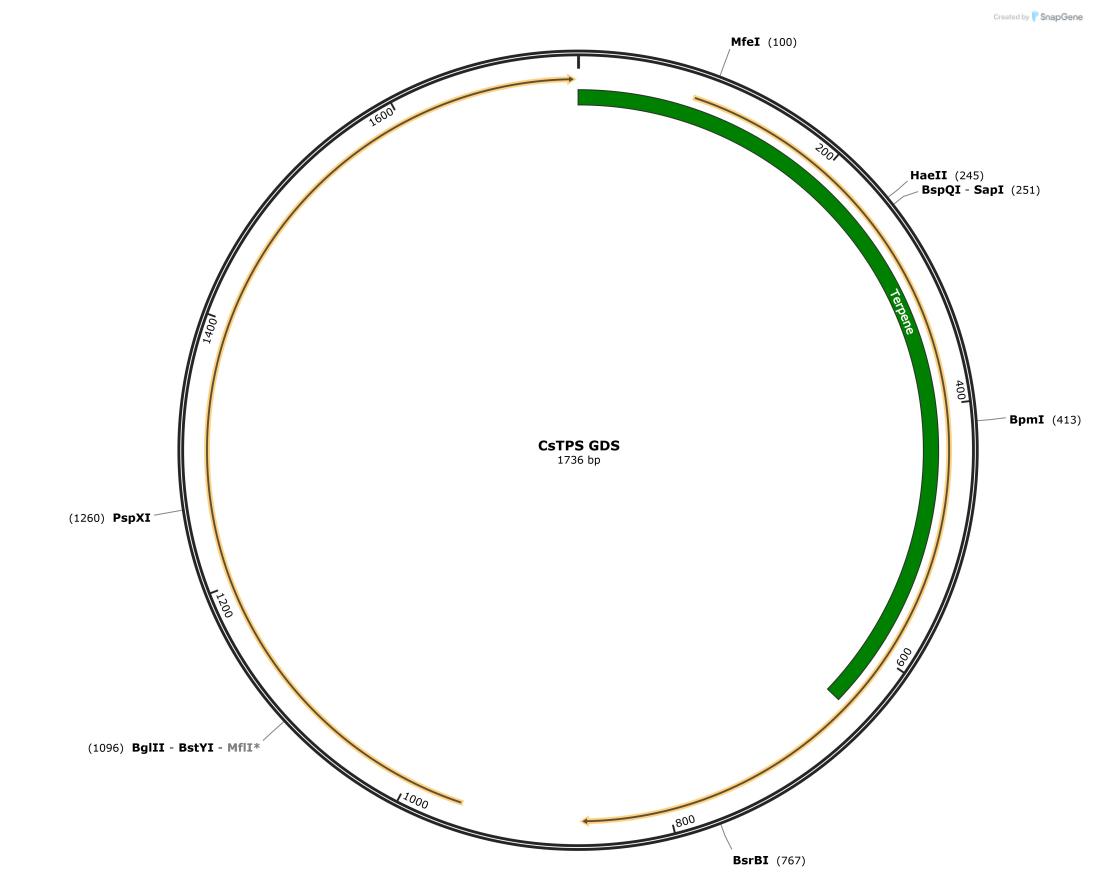


(B)

(A)


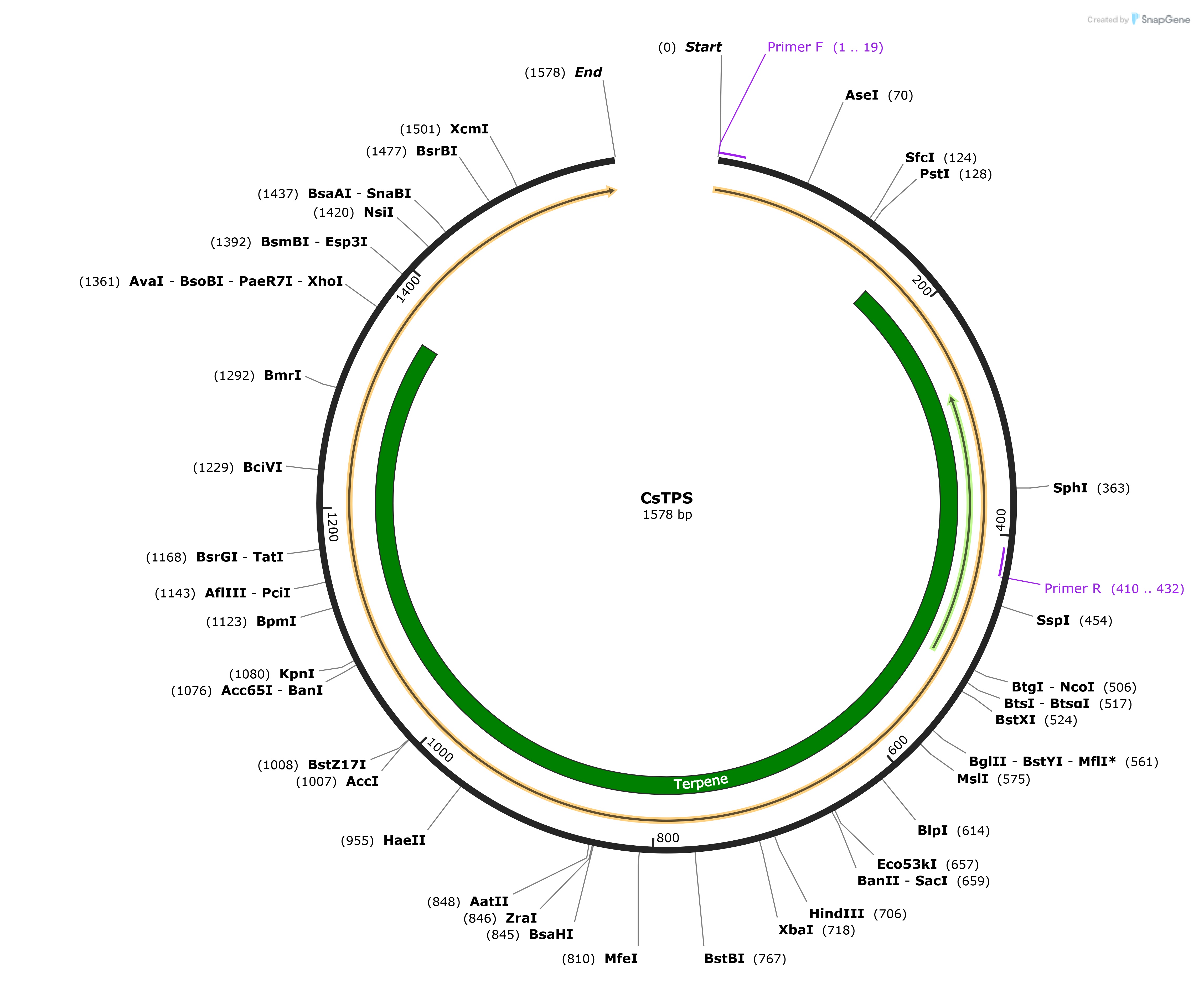


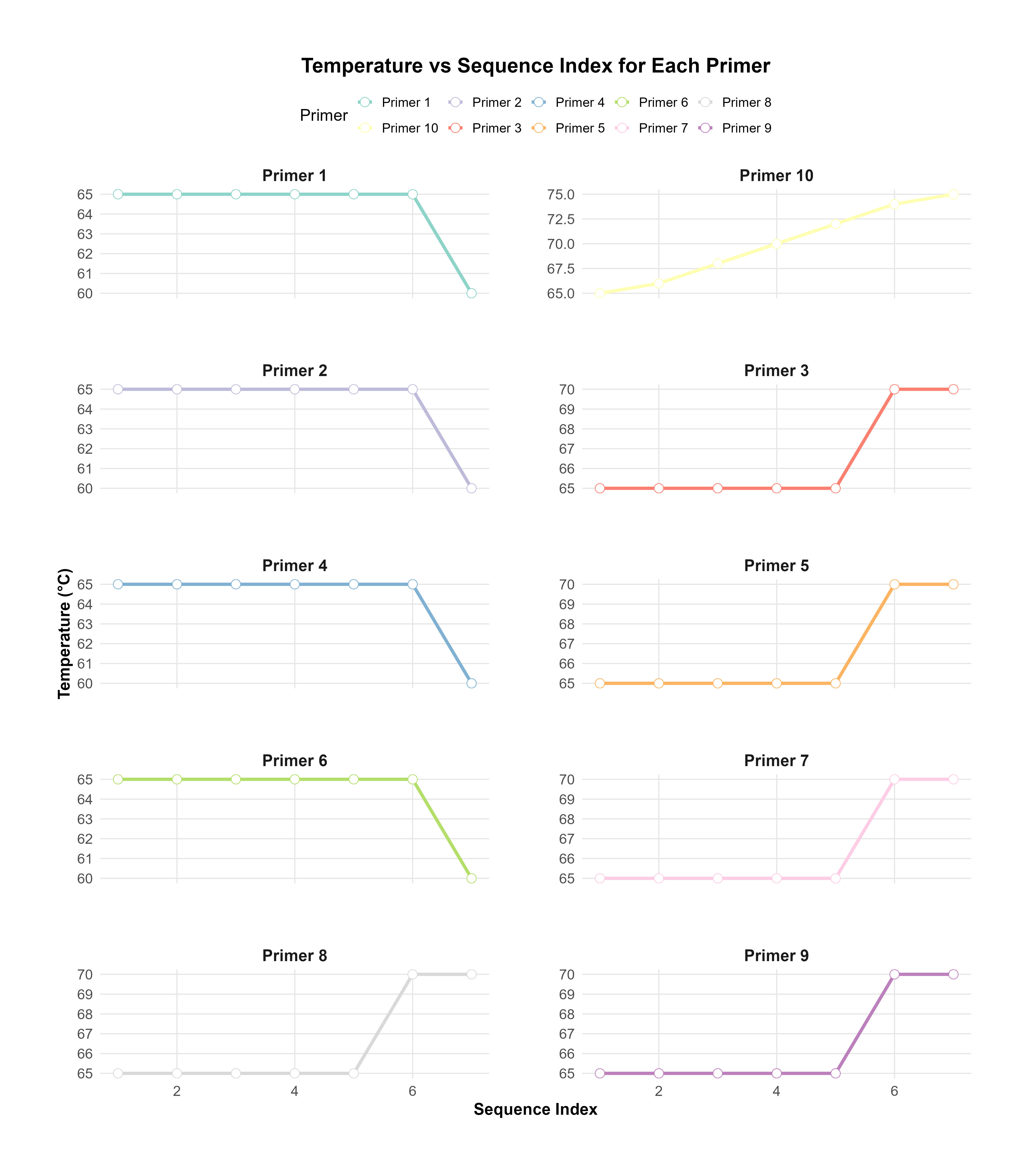


(C)
